# Critical flicker fusion frequency assessment: robustness across photopic illumination in experienced divers

**DOI:** 10.3389/fphys.2026.1824031

**Published:** 2026-09-03

**Authors:** Jochen D. Schipke, Thomas Muth, Anne-Kathrin Brebeck, Dominik von Hertlein, Fabian N. Möller

**Affiliations:** 1 Research Group Experimental Surgery, University Hospital Duesseldorf, Duesseldorf, Germany; 2 Institute of Occupational, Social and Environmental Medicine, Heinrich-Heine-University Düsseldorf, Medical Faculty and University Hospital Düsseldorf, Frankfurt/Main, Germany; 3 Artemis Medizinisches Versorgungszentrum (MVZ) Frankfurt, Frankfurt/Main, Germany; 4 Department of Psychology, German Sport University Cologne, Cologne, Germany; 5 Department of Exercise Physiology, German Sport University Cologne, Cologne, Germany; 6 Department of Aeronauticss and Astroanutics, Massachusetts Institute of Technology, Cambridge, MA, United States; 7 Department of Human Physiology, University of Oregon, Eugene, OR, United States

**Keywords:** arousal, assessment, critical flicker fusion frequency, environment, hyperbarism, illumination

## Abstract

**Introduction:**

Critical flicker fusion frequency (cFFF) is a rapid, noninvasive index of cortical arousal and visual-cognitive performance that has gained renewed interest in diving and other extreme environments. However, the extent to which ambient illumination and water submersion affect cFFF remains insufficiently characterized.

**Methods:**

Nineteen experienced divers (10 females; 24 ± 3 years) completed cFFF testing on land and at 5 m water depth under bright (land: 138 ± 2.4 lx; water: 75 ± 14.8 lx) and dark (0 lx) conditions using a manually operated flicker device. Heart rate (HR) and heart rate variability (HRV) were recorded continuously.

**Results:**

For cFFF, mixed-effects ANOVAs revealed a significant main effect of submersion (p=0.0485) but not illumination (p=0.0754), with higher values in submersion compared to land (+1.4 Hz). Heart rate variability showed a main effect for submersion (p=0.004, +30 ms in submersion) and posthoc differences in dark illumination (p=0.004, +41 ms in submersion). No effects were observed for heart rate or respiration rate. Age showed a low negative correlation with cFFF (r = −0.22 to −0.40) but not with heart rate or heart rate variability. Sex effects were negligible (|r|<0.32).

**Discussion:**

Based on the current 10-minute exposure time, cFFF is robust between the applied illuminations, but small absolute effects were observed from submersion. For human performance and diving research, these findings highlight the importance of baseline measures under comparable environmental conditions to prevent obscuring genuine changes in alertness or cognitive performance. Exposure time and ambient illumination should be standardized or reported to improve comparability across studies and operational environments.

## Introduction

Critical flicker fusion frequency (cFFF) has a long history as a core tool in vision science for its simple administration and straightforward interpretation (Braunstein, 1903). However, the effects of environmental influences like ambient illumination and water submersion on cFFF in diving contexts remain incompletely understood. Following its adoption in the early 20th century, cFFF became a standard paradigm for probing temporal processing in the visual system. More than a century later, cFFF is applied to characterize visual function and to assess disease-related alterations in visual processing in clinical settings (Jiang et al., 2007; Fu et al., 2021) and applied contexts like diving (Balestra et al., 2012; Lafère et al., 2019; Piispanen et al., 2021).

Flicker fusion occurs when temporal changes within the stimulus exceed the visual system’s ability to resolve them, resulting in the perception of a continuous light above the threshold (Davis, 1955). Here, higher cFFF values are associated with increased states of arousal and cognitive awareness (i.e., a conscious understanding of mental processes and the ability to monitor and regulate them in real time) (Smith and Misiak, 1976), as well as greater perceptual accuracy (i.e., the match between perception and the actual stimulus) (Muth et al., 2023). Conversely, lower cFFF values indicate reduced cortical functioning (Curran and Wattis, 2000) and correlate with fatigue and sleepiness, reflecting a diminished state of alertness (Shirane, 2005). Importantly, cFFF values were reported to exhibit notable variability, often attributed to differences in experimental apparatus, methodological approaches, and age. Typical cFFF thresholds show a progressive worsening/reduction of values with increasing age, especially from the age of 60 (Wolf and Schraffa, 1964; Muth et al., 2023).

Quick and accurate assessment of cognitive awareness and perceptual accuracy is especially relevant for diagnostics in extreme and time-sensitive environments. In hyperbaric contexts, cFFF has been applied in hyperbaric chambers (Kot et al., 2015; Tikkinen et al., 2016; Lafère et al., 2019), breath-hold diving (de Asís Fernández et al., 2019), open-circuit diving (Lafere et al., 2010), and rebreather diving (Rocco et al., 2019; Dugrenot et al., 2021) to assess the effects of elevated inspiratory gas pressures on fatigue, cortical arousal, and cognitive function (Hemelryck et al., 2013; Lafère et al., 2019; Piispanen et al., 2021). However, water submersion induces a cascade of physiological adaptations (Bosco et al., 2018), and ambient illumination decreases significantly with water depth. These changes might affect the validity of cFFF in these contexts.

Both illumination and water submersion are expected to modulate cFFF through complementary optical and physiological pathways. In humans, cFFF generally increases with luminance [consistent with Ferry-Porter behavior (Ferry, 1892)], indicating faster temporal integration under brighter conditions, whereas lower light levels slow temporal processing and can reduce cFFF (Rovamo and Raninen, 1988) (Fernandez-Alonso et al., 2023). In operational diving contexts, submersion alters autonomic balance and hemodynamics and has been linked to changes in arousal and cognitive performance, where cFFF is actively evaluated as a practical monitoring tool (Schipke and Pelzer, 2001; Lafère et al., 2019; Vrijdag et al., 2020). Collectively, this literature suggests that cFFF might be lower in reduced illumination and systematically modulated by altered physiology in submersion, underscoring the need to account for these environmental factors when using cFFF to infer alertness or cognitive state in divers.

This study aims to determine the effects of ambient illumination on the critical flicker fusion threshold both poolside (= land) and after submersion in a group of young, healthy divers. We hypothesized (1) that cFFF values would be decreased by lower illumination and (2) increased by water submersion.

## Participants and methods

### Participants

The study included 19 eye-healthy participants (24 ± 3 years, 10 females) who were all certified and experienced divers. A valid medical examination for diving was mandatory for participation. Average height was 175 ± 22 cm, average body mass 72 ± 14 kg, and BMI 23.4 ± 1.4 kg/m^2^.

The investigations were approved by the Ethics Committee of the German Sport University Cologne (100/2023), in accordance with the declaration of Helsinki and its amendments, except for registration in a database (Harriss et al., 2017).

### Materials and methods

All tests were conducted in a well-lit indoor pool (20 m × 20 m × 5 m) with water and air temperatures at 27 and 30 °C, respectively. The diving gear was provided and consisted of a 3 mm wetsuit, a 10 L steel tank with air, a commercially available buoyancy control device (BCD), a breathing regulator, mask, and fins. Some participants used personal masks, and clear vision was ensured for all trials.

The participants were equipped with a Polar V800 sport watch and an H7 chest belt (Polar Electro Oy, Kempele, FI).

### Flicker device

A manually operated flicker device (Scaleo flicker, RP-Engineering GmbH, Esslingen, Germany) was employed in this study due to its ease of use and suitability for underwater use. The device allowed continuous adjustment of flicker frequency via a handwheel. The light source was an LED with a color temperature of 8,000 K (blueish light). The flicker frequency employed a 1:1 duty cycle, i.e., with equal on and off phases, as recommended in the literature (Kelly, 1961; Sekuler et al., 2002). The individual frequency was visible only to the test conductor and was recorded manually.

### Protocol

In a preparatory session, all participants received a comprehensive explanation of the experimental protocol. All participants were presented with demonstrations of both flickering and steady light conditions. Measurements were conducted under bright (land: 138 ± 2.4 lx; submersion: 75 ± 14.8 lx) and dark (= absence of ambient illumination; 0 lx), respectively. A dive mask was worn for conditions in submersion. For clarity, we use the term “submersion” to denote the condition of being submerged in water (5 m depth), distinct from the “land” control condition. The associated diving response refers to the parasympathetic-mediated autonomic reflex triggered by facial water contact and submersion, characterized by bradycardia, increased heart rate variability, and reduced metabolic rate.

In a counterbalanced crossover design, tests were conducted on land and during submersion, kneeling at 5-meter water depth. The stimulus frequency was continuously increased from 20 Hz to the individual cFFF threshold, signaled by the participant. Measures were taken in triplicate by a single test conductor. In cases where participants were uncertain about their decision, or when a clear outlier was observed, all three measurements were repeated. As the distance to the light source was reported to affect cFFF (Hosokawa et al., 1997), participants maintained a fixed distance of 1.5 m to the light source.

### Data preparation and statistics

Average values across the three cFFF measurements at each time point were used for analysis. HR, HRV, and RespR were derived from the ECG signal (Kubios Oy, HRV Scientific 4.1.0, Kuopio, FI) and analyzed as average values over at least 60 seconds of each time point. Data noise affected the extraction of RespR with reductions in the sample size to n = 17 (Land) and n = 16 (submersion).

*Post hoc* power analysis was conducted using G*Power 3.1 (Faul et al., 2009) to evaluate the adequacy of the sample size. With N = 19 participants and a two-way repeated-measures ANOVA design, power was evaluated for detecting small-to-medium effect sizes (f = 0.15 for main effects and interactions).

All statistical analyses were conducted in GraphPad Prism (version 10.6, GraphPad Software, San Diego, USA). Data were first inspected for normality using the Shapiro–Wilk test.

For critical flicker fusion frequency, one-tailed repeated-measures ANOVA (mixed-effects models) with illumination (bright, dark) and submersion (land, submersion) as within-subject factors and subject as a random effect were used with an alpha level of 0.05, based on *a priori* directional hypotheses (submersion increasing cFFF; lower illumination decreasing cFFF). For heart rate, heart rate variability, and respiration rate, two-tailed tests were employed at α = 0.05. Two-tailed equivalents for the cFFF analysis are reported in the Results section. When a significant main effect or interaction was detected, pairwise comparisons with Fisher’s LSD correction were conducted.

Exploratory analyses examining relationships between age, sex, and the physiological variables were performed using Pearson’s correlation coefficients (r) at α = 0.05 to control Type I error risk in hypothesis-generating analyses.

The strength of associations was categorized as very small (|r| < 0.1), small (0.1 ≤ |r| < 0.3), moderate (0.3 ≤ |r| < 0.5), or strong (| r| ≥ 0.5).

All data is presented as individual data points and means ± standard deviations (SD). 95% confidence intervals were reported for pairwise comparisons. Main effects, interaction effects, and significant pairwise comparisons are depicted within figures.

## Results

### Critical flicker fusion frequency

**Figure 1:**
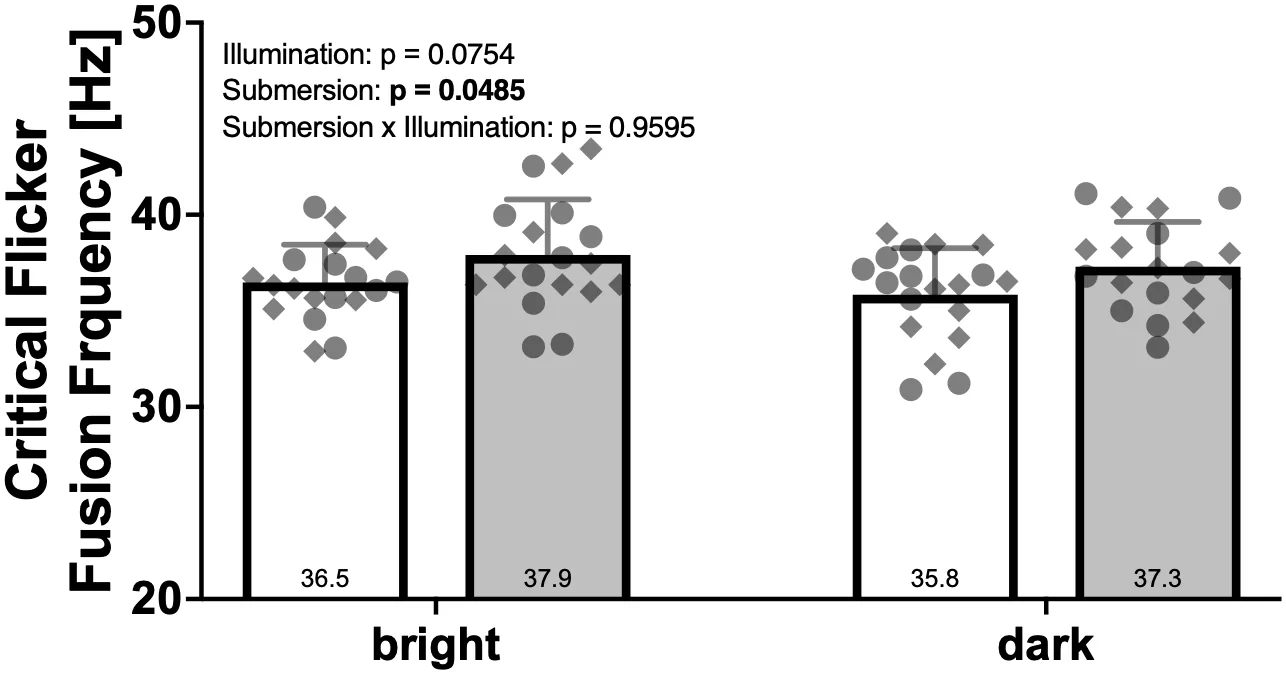
Individual critical flicker fusion frequency (cFFF) during bright and dark conditions on land (light bars, open symbols) and underwater (shaded bars, closed symbols). Females are depicted by open and closed circles and males by open and closed diamonds. If applicable, significant differences are depicted by *.

Significant main effects were observed for submersion (F (1, 36) = 4.173, p = 0.0485 [two-tailed: p = 0.0970], partial η² = 0.10), indicating higher cFFF during water submersion (37.6 ± 2.6 Hz) compared to land (36.2 ± 2.2 Hz) (mean difference: 1.4 Hz; 95% CI: 0.7 - 2.2 Hz; Cohen’s d = 0.54) (**Figure 1**). No effects were observed for illumination (F (1, 36) = 3.352, p = 0.7454) or the interaction between illumination and submersion (F (1, 36) = 0.002615, p = 0.9595). No pairwise comparisons reached significance (**Figure 1**).

### Participant physiological data

**Figure 2:**
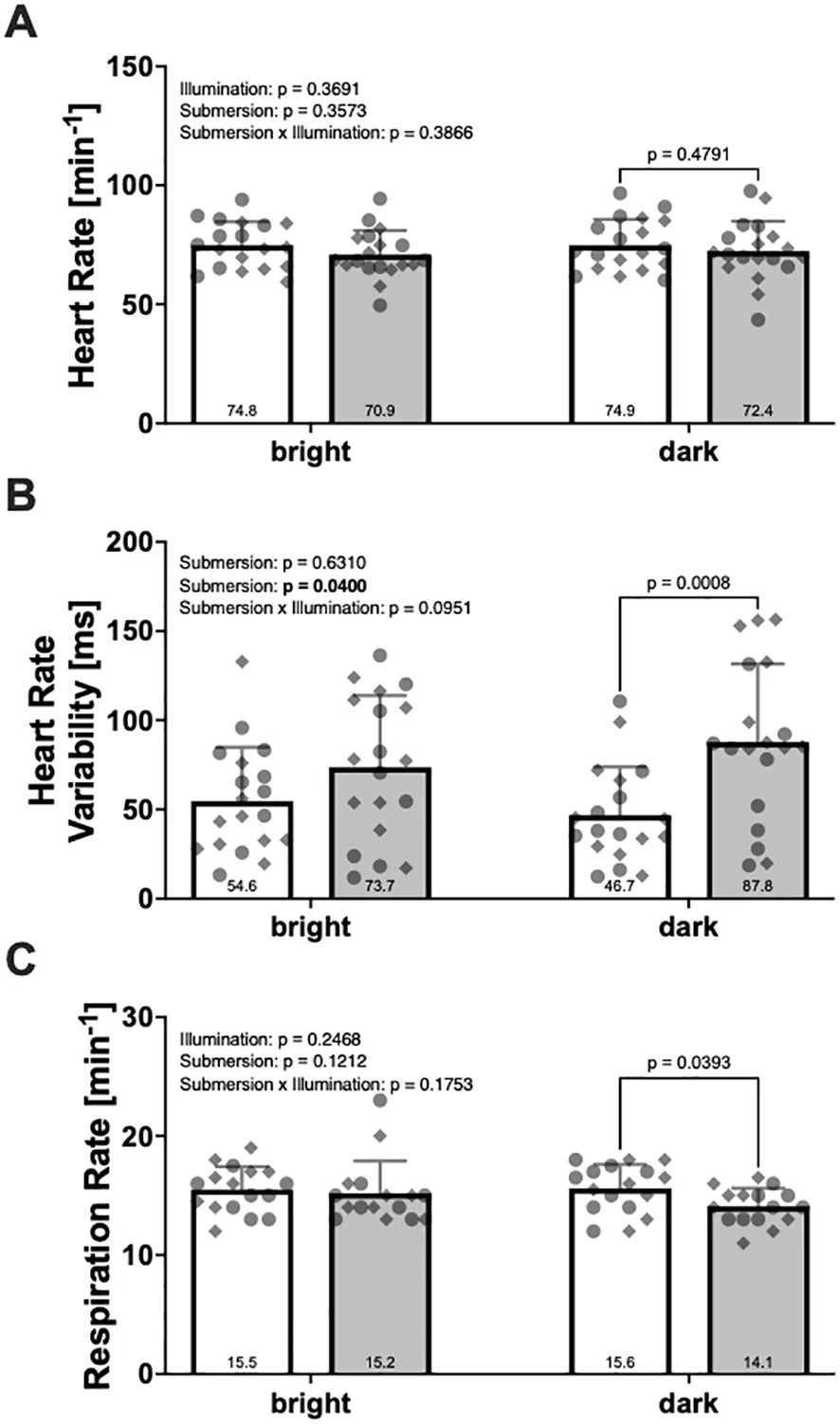
Individual heart rate [HR, **(A)**], heart rate variability [HRV, **(B)**], and respiration rate [RespR, **(C)**] during bright and dark conditions on land (light bars, open symbols) and underwater (shaded bars, closed symbols). Females are depicted by open and closed circles and males by open and closed diamonds. If applicable, significant differences are depicted by *.

No significant main or interaction effects were observed for heart rate (p > 0.05). No difference was observed between values on land (bright: 75 ± 10 min-1; dark: 75 ± 11 min^-1^) and underwater (bright: 71 ± 10 min^-1^, dark: 72 ± 13 min^-1^). The mean difference between bright light and the absence of ambient illumination was -0.72 ± 0.9 min^-1^ (CI: -2.3 to 0.89) (**Figure 2A**).

Heart rate variability showed a significant main effect for submersion (F (1, 36) = 9.481, p = 0.0400, partial η² = 0.21), with mean difference of 30.1 ms (95% CI: 10.5 - 49.7 ms), but not for illumination (p = 0.6310).

Heart rate variability was lower in the dark condition on land (46.7 ± 27.1 ms) compared to submersion (87.8 ± 43.8 ms; mean difference: 41.0 ms; 95% CI: 15.9 - 66.2 ms; p = 0.004 [two-tailed: p = 0.008]), but no differences were detected within the bright condition (mean difference: -19 ms; p = 0.1068) or between different illuminations (both p > 0.1288) (**Figure 2B**).

No significant main or interaction effects were observed for respiration rate (p > 0.05). Respiration rate in the dark condition was lower in submersion than on land (14 ± 2 vs.16 ± 2 min^-1^; mean difference: 1.5 min^-1^; n = 17), while values in the bright condition were indifferent (land: 16 ± 2 min^-1^; submersion: 15 ± 3 min^-1^, mean difference: 0.32; n = 17) (**Figure 2C**).

### Influence of age and sex

Across participants (N = 19), correlations between age and cFFF remained insignificant (all p > 0.09), ranging from r = -0.22 (land bright) to r = −0.40 (land dark). No significant correlations were observed between age and HR or HRV. Correlations between sex and cFFF, HR, and HRV, respectively, remained insignificant.

## Discussion

This study investigated the effects of ambient illumination and water submersion on critical flicker fusion frequency (cFFF) in experienced divers under controlled, near-laboratory conditions.

Regarding optical mechanisms, cFFF was significantly higher during submersion than on land (1.4 Hz; d = 0.54), confirming that submersion influences visual-cognitive processing, while illumination alone showed no significant effect. Heart rate did not significantly change, but autonomic mechanisms were apparent by the substantial increase in heart rate variability, reflecting parasympathetic dominance characteristic of the diving response. Although modest in absolute terms, this within-subject effect was consistent across participants and comparable to magnitudes previously considered operationally relevant in diving research. To contextualize the magnitude of this effect, a 1.4 Hz increase represents approximately 3.8% improvement in cFFF relative to baseline land values. While numerically modest, this change is within the range of variability previously associated with meaningful cognitive or physiological states. For comparison, cFFF declines of 2–4 Hz have been documented in nitrogen narcosis at moderate depths (Lafère et al., 2019) and acute fatigue or reduced alertness typically produces declines of 1–3 Hz (Muth et al., 2023). Thus, the observed submersion-associated increase of 1.4 Hz suggests that submersion may partially counteract reductions in cFFF that would otherwise occur during hyperbaric exposure or fatigue states. In operational diving contexts, where rapid assessment of alertness is critical, even modest shifts in cFFF consistent with parasympathetic activation may be physiologically meaningful despite their small absolute magnitude.

The observed differences were small in absolute terms but consistent across participants, suggesting that cFFF is relatively robust to moderate illumination changes (0–138 lx), yet sensitive to parasympathetic-mediated submersion-related physiological changes. While heart rate did not decrease during submersion in the present study, the concomitant increase in heart rate variability nevertheless suggests enhanced parasympathetic-mediated activity, consistent with partial activation of the also parasympathetic-driven diving response (Bosco et al., 2018). It should be noted that the submersion effect on cFFF approaches but does not reach conventional two-tailed significance (p = 0.097), reflecting the borderline nature of this finding. While our *a priori* directional hypothesis justified one-tailed testing, this result warrants cautious interpretation and validation in larger samples.

Together, these findings demonstrate that environmental context and parasympathetic-mediated autonomic regulation can subtly alter visual-cognitive perceptual thresholds, underscoring the importance of standardizing illumination and submersion conditions when using cFFF to assess arousal or cognitive performance in diving and comparable operational environments.

The cFFF increase during submersion likely reflects optical or neural mechanisms related to water properties (e.g., altered light scattering, refractive changes, or proprioceptive feedback), rather than autonomic effects. In contrast, the significant increase in heart rate variability during submersion reflects clear autonomic activation, specifically parasympathetic predominance, as evidenced by the diving reflex. Both illumination conditions (0–138 lx) remained within the photopic luminance range, where cone-driven vision predominates and temporal sensitivity is robust. The absence of an illumination effect reflects cFFF stability within the photopic domain rather than universal light insensitivity. Notably, ambient lux does not directly equal retinal illuminance (trolands), which also depends on stimulus luminance, pupil diameter, and optical geometry. Pupil measurements were not obtained, limiting our ability to calculate actual retinal illuminance or determine whether pupils adapted differently between conditions. Within the photopic range tested here, modest lighting changes are therefore unlikely to produce meaningful cFFF differences.

Our findings align with previous human studies showing that cFFF varies most strongly across extreme luminance transitions, such as from daylight to mesopic or scotopic levels (Kelly, 1961; Fernandez-Alonso et al., 2023), thus indicating that cFFF measurements are reliable across moderate light variations, reinforcing their suitability for operational use in diving and other controlled environments. However, larger gradients, such as between daylight and greater water depths or nightdives, may still reduce flicker sensitivity by shifting toward rod-dominant vision (Fernandez-Alonso et al., 2023). Future studies could extend this work by manipulating stimulus luminance and adaptation time to reach truly mesopic or scotopic conditions, or by reporting retinal illuminance (trolands) to more precisely link light exposure with visual performance outcomes.

Submersion significantly affected cFFF, with slightly higher thresholds observed underwater compared to land. This finding suggests that submersion-induced physiological changes, rather than optical factors, might have influenced cFFF. Water submersion induces central hypervolemia and stimulates baroreceptor and trigeminal afferents, resulting in a reflex increase in parasympathetic activity and reduced sympathetic tone (Schipke and Pelzer, 2001; Pendergast et al., 2015). While heart rate did not decrease during submersion in the present study, the concomitant increase in heart rate variability nevertheless suggests enhanced parasympathetic activity, consistent with partial activation of the classical diving response (Bosco et al., 2018). This slight cFFF increase during submersion could therefore reflect a shift toward a calm physiological state, characterized by parasympathetic-mediated dominance but preserved visual-cognitive cortical arousal. Previous studies have shown that mild parasympathetic-mediated activation or improved cerebral perfusion can maintain or even enhance visual-cognitive processing and cognitive stability under moderate physiological stress (Balestra et al., 2018; Lafère et al., 2019). Conversely, excessive parasympathetic dominance or hypoxia tends to lower cFFF by reduced alertness (Vrijdag et al., 2020).

Taken together, these results demonstrate that submersion introduces subtle but meaningful effects on visual-cognitive perceptual thresholds, likely mediated through cardiovascular and parasympathetic-mediated autonomic adjustments rather than through direct optical or environmental influences. This emphasizes the need to control or document submersion-related physiological states, such as heart rate variability or breathing pattern, when using cFFF as an indicator of arousal or cognitive function in operational contexts underwater.

### Limitations

A range of factors can influence cFFF measurements, many of which extend beyond the illumination and submersion parameters tested in the present study. Classic work by Erlick and Landis (1952) identified over ten potential confounders, including the color, size, contrast, and duty cycle of the flicker stimulus which increase methodological variability. More recent reviews emphasize that stimulus intensity, eccentricity, and light-dark ratio can alter thresholds, while interindividual differences in adaptation state, pupil size, and fatigue introduce additional variability (Mankowska et al., 2021; Muth et al., 2023). Beyond optical parameters, biological and temporal factors such as age, circadian rhythm, and core body temperature also modulate temporal processing (Mankowska et al., 2021).

Individual cFFF values in our healthy cohort ranged from 33–42 Hz, yielding intra-individual standard deviations of 1–3 Hz across repeated trials; thus, our observed 1.4 Hz effect, while consistent across participants, approaches the magnitude of measurement variability itself, warranting cautious interpretation in clinical settings. Pupil diameter was not measured, preventing calculation of retinal illuminance (trolands) and assessment of pupil-mediated adaptation differences between conditions.

Potential operator bias due to manual adjustment of the flicker device and possible familiarization effects across repeated trials should also be considered. Although a learning effect cannot be excluded entirely, the counterbalanced crossover design, where participants were randomly assigned to different sequences of conditions, makes a systematic bias toward any specific illumination or submersion condition unlikely.

Several specific design constraints merit discussion. The illumination range (0–138 lx) represents moderate light levels typical of controlled pool environments. Although adequate for reproducibility, it does not encompass the full photopic-mesopic transitions of open-water dives. The “dark” condition represented an absence of ambient illumination (0 lx) rather than true scotopic darkness; the ~10-minute adaptation period was sufficient for partial cone adaptation but insufficient for full rod dominance, and the LED stimulus itself provided additional light that prevented complete dark adaptation. This design choice reflects the practical constraints of underwater testing but limits generalizability to truly mesopic or scotopic conditions. Finally, while submersion depth was shallow (5 m), differences in hydrostatic pressure and gas partial pressures may still have contributed minor effects. Based on prior hyperbaric studies, however, the small increase in pN_2_ (~1.1 bar) and PiO_2_ (~0.42 bar) at this depth would be far below levels known to impair cFFF (Kot et al., 2015).

### Conclusions

The current findings demonstrate that cFFF is relatively stable across moderate photopic illumination levels and submersion conditions within the applied range and duration of exposure (0–138 lx, ~10 min), supporting its reliability for assessing cortical arousal and perceptual performance in aquatic and operational research. The combined use of cFFF with physiological markers such as heart rate variability or cerebral oxygenation could strengthen interpretation by distinguishing between sensory and autonomic influences. For applied practice, these findings indicate that cFFF testing can be conducted reliably without strict control of ambient light, provided that conditions remain photopic and consistent within subjects. Future studies should quantify retinal illuminance (trolands), extend adaptation periods, and include a wider range of luminance and depth conditions to refine predictive models. Ultimately, establishing standardized testing protocols will enhance the utility of cFFF as a quick, non-invasive index of human alertness, fatigue, and performance stability under environmental stress.

## Data Availability

The original contributions presented in the study are included in the article/supplementary material. Further inquiries can be directed to the corresponding author.
